# 

**Published:** 1896-11

**Authors:** 


### EDITORIAL.

### The National Association of Dental Faculties.

The time and place of holding the next meeting of the Faculties' Association is now receiving a good deal of attention on the part of the Faculties of the various Colleges. Nearly all concede the desirabilty, in some respects at least, of a change of time for the meeting, quite a number advocating the holiday time as the most feasible; others preferring it to be held in April, at the time when quite a number of colleges have a few days' vacation; and other are strongly opposed to any change of time.

On one point all seem to be of about the same opinion, viz: That more time ought to be given to the work of the Association than it has received heretofore; that the importance of its work is becoming more important every year, and better opportunity ought to be afforded for it; its work has, each year, been crowded and rushed along with too great haste. The educational interests of the dental profession is, in a large measure, in the hands of this body, and while it has done grand work in the past, it must not be forgotten that great and rapid progress is being made in all educational work, and no department can afford to fall behind.

Many subjects that have not, as yet, been before this body at all, ought to have earnest consideration and adjustment; these will occur to the minds of those interested without mention here. It is to be hoped that if the time of holding the meeting is changed that it will be accepted by all, and a full attendance be had. Six sessions, occupying two days and evenings, devoted strictly to business, would accomplish much more than has ever been done at any one meeting.

An effort is now being made to ascertain the wish of the membership, and if it is decided to hold a meeting in mid-winter or in the spring, it is to be hoped that the largest possible number will be present; and we suggest that each one have in mind some important subject for consideration; indeed, a program of subjects ought to be prepared and distributed to all the members a month or two before the meeting, whenever it may be held. Looking toward that point the suggestion is here made, that the various members formulate questions and subjects, and forward them to the Executive Committee, that a program may be prepared. Such preparations should be made for each and every meeting whenever it may be held.

### Ohio State Dental Society.

December 1, 2 and 3, 1896.

PROGRAM.

President's Address, Dr. Henry Barnes, Cleveland.

"Dental Legislation," Dr. G. H. Wilson, Cleveland. Discussers, Drs. J. Taft, C. I. Keely, A. F. Emminger.

"Teeth Extracted without Pain," Dr. W. I. Jones, Nelsonville. Discussers, Drs. H. S. Ainsworth, W. H. Sedgwick, F. O. Jacobs.

"Dental Operations During Pregnancy." Discussers, Drs. H. A. Smith, C. R. Butler, E. H. Raffensperger.

"The Use of Gold and Tin in Combination as a Filling Material," Dr. W. B. Connors, Akron. Discussers, Drs. A. E. McConkey, Ira Brown, A. T. Whitesides.

"Cataphoresis—A Summary of my Experience with Cataphoresis," Dr. L. L. Barber, Toledo.

"The Galvanic Current," Dr. H. G. Husted, Oberlin.

"Some Reasons why we Fail with Cataphoresis," Dr. Wm. H. Hirsch, Piqua.

"Bleaching by Cataphoresis—Clinic," Dr. Henry Barnes, Cleveland. Discussers, Drs. L. E. Custer, P. S. Bollinger, J. R. Bell.

"Smooth-Pointed Pluggers," Dr. Chas. G. Myers, Cleveland. Discussers, Drs. W. H. Todd, E. Sloane, C. R. Converse.

"Empyemia of the Antrum," Dr. John T. Stephan, Cleveland. Discussers, Drs. Grant Mollyneaux, M. H. Fletcher, F. W. Sage.

"Bacteria of the Mouth," Dr. C. T. Whinery, Toledo. Discussers, Drs. L. P. Bethel, H. F. Harvey, H. R. Clark.

"Imagination," Dr. J. S. Cassedy, Covington. Discussers, Drs. C. M. Wright, E. G. Betty, D. W. Clancy.

"Antiseptics and Disinfectants in Office Practice," Dr. H. B. Bartilson, Columbus. Discussers, Drs. S. B. Dewey, David Harroun, J. A. Lupton.

The meeting will be held at the Neil House where a rate of $2.50 per day will be given to those in attendance.

**A MONTHLY MAGAZINE DEVOTED TO THE INTERESTS OF THE**

### DENTAL PROFESSION.

*Terms Reduced to* $2.00 *Per Year. Single Copies,* 25 *Cents.*

**EDITED BY PROF. J. TAFT, D.D.S.**

—AND—

**Published by SAM'L A. CROCKER & CO., Ohio Dental and Surgical Depot.**

The volume commences in January and closes in December.

The REGISTER being issued monthly is always fresh, therefore Indispensable to the practitioner who desires to keep posted up with the new inventions and improvements which are daily developed. Neither expense nor effort will be spared to give value and variety to its contents. Articles will be illustrated with well executed engravings whenever they will add to the interest or assist to explanation.

Its extensive circulation and frequency of issue make it one of the BEST DENTAL ADVERTISING MEDIUMS in the United States.

All subscriptions must begin with January or July.

Local or special notices, five lines or less, will be inserted for $3 each insertion; over five lines, 50 cts. per line.

All advertising bills payable in advance, or at furthest, after the issue of the first number containing the advertisement. All transient advertisements to be paid for in advance.

☞CONTRIBUTIONS SOLICITED.

Exclusively Editorial Communications should be addressed to the Editor.

The latest number of the REGISTER may be ordered with or without other goods at any time, and all letters should be addres[illegible]

